# Neuroendocrine-vascular interaction is associated with thalamocortical disorganization and cognitive decline in perimenopausal hypertension

**DOI:** 10.3389/fmed.2026.1775371

**Published:** 2026-02-27

**Authors:** Taipeng Zeng, Xiaoyang Wang, Yuhang Zhang, Minghui Mao, Chengkun Hong, Zhen Yu, Hao Huang, Jianfeng Chu, Liyuan Fu

**Affiliations:** 1 College of Integrative Medicine, Fujian University of Traditional Chinese Medicine, Fuzhou, China; 2 Fuzong Teaching Hospital, Fujian University of Traditional Chinese Medicine (900th Hospital), Fuzhou, China

**Keywords:** cognitive impairment, hypertension, morphometric similarity gradient, neuroendocrine-vascular interaction, perimenopause, thalamic subregions

## Abstract

**Background:**

Hypertension (HTN) during perimenopause significantly increases the risk of cognitive decline. However, how hemodynamic stress and hormonal fluctuations (e.g., estradiol depletion) interact to reshape the brain’s macroscale organization remains poorly understood. This study aimed to map the “hormone-thalamus-cortex” axis to explore these mechanisms.

**Methods:**

Thirty perimenopausal women with HTN and 30 healthy controls (HC) underwent 3-T MRI, blood pressure monitoring, and serum hormone analysis. We constructed individual cortical morphometric similarity (MS) gradients and segmented the thalamus into 52 subregions to analyze the interplay between hormones, brain structure, and cognition.

**Results:**

We found that the principal MS gradient exhibited a distinct “stretching” pattern in the HTN group compared with HC, characterized by a significant positive spatial correlation with the HC gradient. The HTN group exhibited significantly elevated gradient values in the Visual and Somatomotor networks (VIS and SMN), as well as in specific regions within the frontoparietal network (FPN, left parietal subregion-4) and ventral attention network (VAN, right medial subregion-5). Thalamic analysis revealed a bidirectional remodeling pattern: atrophy in sensory/executive nuclei (e.g., VPL, LGN, MDm) and hypertrophy in intralaminar nuclei (e.g., CL, AV). Critically, estradiol (E2) depletion and FSH elevation were linked to specific thalamic atrophy (e.g., Right Pc), which in turn predicted lower MMSE scores and VAN disruptions.

**Conclusion:**

Perimenopausal HTN is associated with a neuroendocrine-modulated reorganization of the brain’s hierarchy. The findings suggest that hormonal shifts exacerbate thalamic vulnerability, potentially contributing to cortical instability and cognitive impairment. This identifies perimenopause as a critical window for integrated vascular and hormonal interventions to preserve cognitive health.

## 1 Introduction

Hypertension (HTN) is a major modifiable risk factor for cognitive impairment and dementia, posing a significant global health burden (1, 2). Chronic blood pressure elevation damages the cerebrovascular system, leading to white matter hyperintensities, cortical thinning, and network disruptions that impair executive and memory functions (3–6). Despite these known risks, how the “stress” of HTN reshapes the brain’s macroscale hierarchical organization—particularly before the onset of clinical dementia—remains poorly understood.

Epidemiological evidence indicates a sexual dimorphism in HTN-related brain injury, with women experiencing more rapid cognitive decline during the menopausal transition (7–10). This period is characterized by dramatic fluctuations in 17β-estradiol (E2) and follicle-stimulating hormone (FSH). Given estrogen’s neuroprotective roles in vascular health and synaptic plasticity (11, 12), perimenopausal women with HTN may face a “double-hit” of vascular pathology and hormonal depletion. However, the specific brain circuits vulnerable to this interaction remain under-investigated.

The thalamus serves as a critical hub for coordinating cortical activity and supporting high-level cognition (13–15). Recent evidence suggests the thalamus is a heterogeneous collection of nuclei with varying sensitivities to vascular and endocrine stress (16–18). Traditional volumetric studies often treat the thalamus as a single unit, which may mask subregion-specific changes. For example, high-metabolic sensory nuclei may be more sensitive to hypoperfusion, while limbic-associated nuclei might respond differently to hormonal shifts. Therefore, a fine-grained thalamic analysis is essential for understanding the brain’s response to perimenopausal HTN.

Cognitive health relies on the efficient integration of information across the brain’s hierarchy, which can be characterized using morphometric similarity (MS) gradients (19–21). MS gradients integrate multiple MRI features to reflect the organizational axis from sensory to transmodal areas (22, 23). While MS gradients have revealed disruptions in various brain disorders (24–27), it remains unknown how HTN and hormonal aging together impact this hierarchy or whether these changes originate from thalamic degradation. Furthermore, genetic factors are also known to shape brain networks, particularly connections that are functionally important and metabolically costly (28, 29). The Allen Human Brain Atlas (AHBA) provides a comprehensive resource to link regional gene expression to macroscale brain organization (30). However, it remains unclear how the specific cortical morphological alterations observed in perimenopausal HTN relate to underlying gene expression patterns.

**FIGURE 1:**
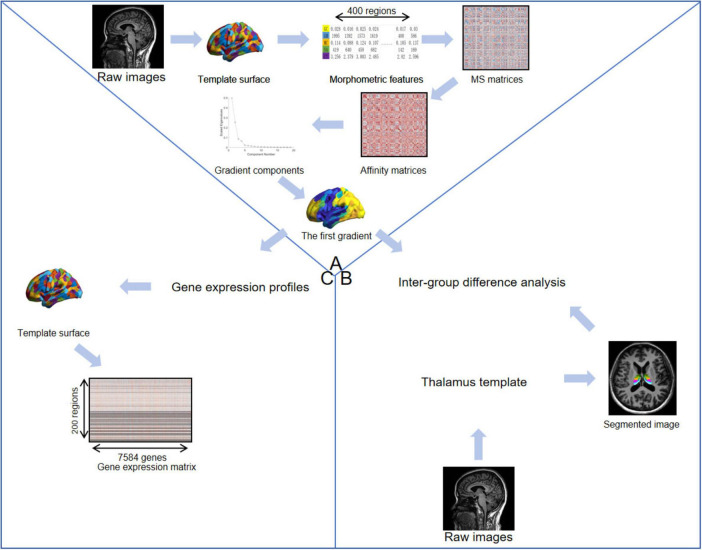
Schematic illustration of the research methodology. **(A)** Gradient construction. First, morphological features were generated from individual structural images. Regional morphological profiles were extracted based on the Schaefer 400 atlas, and the features for each region were concatenated into vectors. MS matrices were constructed for each subject by calculating the Pearson correlation between the vectors of every pair of regions. Affinity matrices were then created by applying a kernel function to the MS matrices. Finally, the first gradient map for each subject was obtained by decomposing the affinity matrix using diffusion map embedding. **(B)** Thalamic subdivision segmentation. Thalamic subdivisions were segmented based on a probabilistic atlas of the human thalamic nuclei. **(C)** Gene expression. Gene expression values for each gene within each brain region (left hemisphere only) were extracted from the AHBA database to construct a gene expression matrix [adapted from (26, 32)].

In the present study, we employed a multimodal approach integrating cortical MS gradient mapping, high-resolution thalamic parcelation, and imaging-transcriptomic association analysis to systematically investigate the neuroanatomical and functional alterations in perimenopausal women with primary HTN. We hypothesized that: (1) Perimenopausal HTN would induce a bidirectional remodeling of thalamic subregions rather than uniform atrophy; (2) This thalamic remodeling would propagate upward, leading to disruptions in the cortical sensory-transmodal hierarchy; and (3) These multi-level disruptions are likely driven by the “double-hit” of hemodynamic stress and hormonal fluctuations (specifically E2 withdrawal and FSH elevation), and we further explored whether these patterns are associated with the underlying transcriptomic architecture defined by specific gene expression profiles. An overview of the analytical framework is summarized in Figure 1.

## 2 Materials and methods

### 2.1 Patients

This study was approved by the Biomedical Ethics Committee of the 900th Hospital of the Joint Logistics Support Force, and written informed consent was obtained from all participants. Based on the strict inclusion criteria, a total of 30 perimenopausal women with primary HTN were recruited from the 900th Hospital of Joint Logistics Support Force, and 30 healthy control (HC) women from the local community. For the HTN group, participants were required to have a confirmed diagnosis of essential hypertension in accordance with the 2024 Chinese Guidelines for Prevention and Treatment of Hypertension—defined as an office systolic blood pressure (SBP) ≥ 140 mmHg and/or diastolic blood pressure (DBP) ≥ 90 mmHg measured on three separate days without medication, or the current use of antihypertensive pharmacotherapy. Additionally, eligibility was restricted to females aged 45–55 years to specifically target the perimenopausal transition. Participants exhibited clinical features consistent with the perimenopausal transition, defined as either menstrual cycle irregularity or the presence of symptoms such as hypomenorrhea and irritability despite regular cycling. Furthermore, patients were required to have a hypertension disease duration of less than 2 years to minimize the confounding effects of long-term chronic vascular remodeling on brain structure. Conversely, the HC group was required to have an SBP < 120 mmHg and DBP < 80 mmHg, with no history of hypertension or antihypertensive medication use. All participants provided written informed consent and demonstrated willingness to cooperate with the study procedures. The exclusion criteria were as follows: a history of stroke, dementia, schizophrenia, epilepsy, Parkinson’s disease, bipolar disorder, or any other major neurological or psychiatric disorders; use of medications affecting cognition; claustrophobia; secondary HTN; end-stage heart disease; renal failure (including dialysis); diabetes mellitus; or atrial fibrillation.

### 2.2 Clinical and neuropsychological assessments

Clinical and neuropsychological assessments All participants underwent a comprehensive clinical evaluation. Blood pressure measurements strictly adhered to standard protocols. SBP and DBP were measured at three distinct time points: prior to neuropsychological testing, before blood biochemical examination, and before MRI scanning. The final reported blood pressure values represent the average of these three measurements. For hormonal analysis, fasting venous blood samples were collected from all subjects following a fasting period of more than 8 h. Serum levels of sex hormones were measured, including FSH, luteinizing hormone (LH), E2, progesterone (PROG), testosterone (T), and prolactin (PRL). Additionally, cognitive function was assessed using the Mini-Mental State Examination (MMSE) and the Montreal Cognitive Assessment (MoCA) by a trained neuropsychologist.

### 2.3 Neuroimaging data acquisition and preprocessing

All images were acquired using a United Imaging uMR 770 3.0T superconducting MRI scanner equipped with a 32-channel phased-array head coil. Routine sequences were initially performed to screen for and exclude brain lesions, including tumors, cerebral hemorrhage, chronic cerebral infarction, and congenital intracranial abnormalities. High-resolution whole-brain 3D T1-weighted structural images (T1WI-3D) were acquired using a fast gradient echo sequence with the following parameters: repetition time (TR) = 6.94 ms; echo time (TE) = 3 ms; slice thickness = 1 mm; interslice gap = 0 mm; field of view (FOV) = 256 mm × 256 mm; matrix size = 256 × 256; 176 sagittal slices; and acquisition time = 269 s. Following image acquisition, the 3D high-resolution T1-weighted images were preprocessed for cortical surface reconstruction using FreeSurfer (v8.1.0^1^) (31). The preprocessing pipeline included skull stripping, tissue segmentation, surface reconstruction, and spherical normalization parameter estimation.

### 2.4 Thalamic parcelation

The thalamus was segmented into 52 subregions (26 per hemisphere) using the probabilistic atlas of human thalamic nuclei (32). Steps were as follows: (i) individual T1 images were registered to MNI152 space (1 mm isotropic) using FSL^2^ (33); (ii) the thalamic atlas was inversely transformed to subject space; (iii) atlas labels were overlapped with subject-specific thalamic masks from FreeSurfer to yield voxel-wise parcellations; (iv) all outputs were visually inspected to ensure accuracy.

### 2.5 Construction of MS gradients

The cortical surface was parcellated into 400 regions using the Schaefer atlas (34). Five morphometric features—gray matter (GM) volume, cortical thickness (CT), surface area (SA), intrinsic (Gaussian) curvature (IC), and mean curvature (MC)—were extracted per parcel, z-scored, and correlated to build a 400 × 400 MS matrix for each subject. Gradient decomposition was performed using the BrainSpace toolbox^3^ (35). This study focused on the principal gradient, which reflects the sensory–association axis and is sensitive to disease effects (36). Group-level gradients were aligned across subjects for statistical comparison.

### 2.6 Gene expression data preprocessing

Gene expression data were obtained from the AHBA database (37), derived from six postmortem brains (including 3,702 independent samples). We used the abagen toolbox^4^ (38) to process the transcriptome data. Since the AHBA database provides complete genomic coverage primarily for the left hemisphere (data from all six donors), whereas right hemisphere data are available for only two donors, we restricted the transcriptomic-neuroimaging association analysis to the left hemisphere (200 regions) to ensure the stability and reliability of the gene expression profiles. The preprocessing of gene expression data involved nine steps: (i) updating probe-gene annotations; (ii) intensity-based filtering; (iii) probe selection; (iv) sample-brain region matching; (v) missing data handling; (vi) sample-level normalization; (vii) gene-level normalization; (viii) calculation of sample-brain region combination metrics; (ix) selection of stable genes. A total of 7,584 genes were retained finally. Ultimately, the gene expression matrix used for subsequent analyses was 200 brain regions × 7,584 genes.

### 2.7 Transcription-neuroimaging association analysis

Associations between gene expression and MS gradient differences were assessed using partial least squares (PLS) regression (39). The predictor was the z-scored gene expression matrix (200 regions × 7584 genes), and the response was the regional t-map of case–control gradient differences (restricted to the left hemisphere). Permutation testing with spatial spin (10,000 iterations) evaluated the significance of PLS components (40, 41). Genes contributing significantly (*p* < 0.001, BH-FDR corrected) were retained for enrichment analyses.

### 2.8 The MS gradient comparison

Between-group differences in regional gradient scores were tested using general linear models (GLMs). To contextualize alterations, mean gradient values were computed within Yeo functional networks (42). Multiple comparisons were corrected at the regional level [*p* < (1/n) = 0.0025, where *n* = total regions] and Benjamini-Hochberg false discovery rate (BH-FDR) at the network level (*p* < 0.05).

### 2.9 Thalamic subregional volumetric analysis

A GLM was used to assess group differences across the 52 thalamic subregions, with total intracranial volume (TIV) included as a covariate. Volumetric data and TIV were estimated via FreeSurfer (v8.1.0, see text footnote 1) (31). Given the exploratory nature of this study and the relatively small sample size, applying strict multiple comparison corrections was deemed overly conservative and could increase the risk of Type II errors (false negatives). Therefore, we reported uncorrected *p*-values (*p* < 0.05) to maintain sensitivity for detecting subtle thalamic remodeling patterns. We interpret these findings as hypothesis-generating, warranting validation in larger cohorts.

### 2.10 Correlations

Partial correlation analyses were performed to assess associations between clinical variables and neuroimaging metrics exhibiting significant group differences (i.e., principal MS gradient and specific thalamic subregions) in the HTN group, controlling for TIV. As these analyses were hypothesis-driven and restricted to significant regions, uncorrected *p*-values are reported.

### 2.11 Statistical analysis

Demographic and clinical characteristics were compared between groups using independent two-sample *t*-tests for normally distributed continuous variables and Mann-Whitney U tests for non-normally distributed variables. A two-tailed *p*-value < 0.05 was considered statistically significant.

## 3 Results

### 3.1 Data samples

The HTN and HC groups were well-matched for age (*z* = −0.396, *p* = 0.692) and TIV (*t* = 1.045, *p* = 0.300). However, the HTN group exhibited significantly higher systolic (132.77 ± 13.60 mmHg) and diastolic (86.77 ± 10.87 mmHg) blood pressure compared to controls (both *p* < 0.001). Crucially, hypertensive participants demonstrated marked cognitive impairment, evidenced by significantly lower scores on both the MMSE (*z* = −4.294, *p* < 0.001) and MoCA (*z* = −5.467, *p* < 0.001) assessments. Additional demographic and clinical characteristics are summarized in Table 1.

**TABLE 1:** Comparison of demographics, imaging metrics, blood pressure, and laboratory results between the two groups (mean ± SD/median (Q1, Q3).

Characteristics	HTN group	HC group	*t*/*z*-value	*P*-value
Age (years)	48.5 (45.75, 54)	48.5 (45, 52.25)	−0.396^a^	0.692
TIV (cm^3^)	1390.43 ± 124.25	1358.55 ± 111.65	1.045	0.3
SBP (mmHg)	132.77 ± 13.60	115.97 ± 8.82	5.678	<0.001
DBP (mmHg)	86.77 ± 10.87	74.70 ± 7.59	4.983	<0.001
AHM (*n*, %)	30 (100%)	0 (0%)	NA	NA
FSH (mIU/mL)	7.18 (4.25, 53.05)	19.91 (5.41, 66.32)	−1.057^a^	0.29
LH (mIU/mL)	9.32 (3.37, 27.92)	12.72 (4.18, 32.21)	−0.473^a^	0.636
E2 (pg/mL)	48.25 (0.50, 172.68)	45.37 (14.50, 84.85)	−0.134^a^	0.894
PROG (ng/mL)	0.39 (0.26, 1.15)	0.46 (0.32, 10.47)	−0.828^a^	0.408
T (ng/mL)	0.30 ± 0.16	0.31 ± 0.14	−0.316	0.753
PRL (ng/mL)	11.04 (8.17, 14.61)	10.68 (8.36, 14.62)	−0.274^a^	0.784
MMSE (score)	29.00 (28.00, 30.00)	30.00 (30.00, 30.00)	−4.294^a^	<0.001
MoCA (score)	27.00 (26.00, 28.00)	29.00 (28.00, 30.00)	−5.467^a^	<0.001

^a^Represents *z*-values obtained from the Mann-Whitney U test. HTN, hypertension; HC, healthy control; TIV, total intracranial volume; SBP, systolic blood pressure; DBP, diastolic blood pressure; AHM, antihypertensive medication; FSH, follicle-stimulating hormone; LH, luteinizing hormone; E2, estradiol; PROG, progesterone; T, testosterone; PRL, prolactin; MMSE, Mini-Mental State Examination; MoCA, Montreal Cognitive Assessment.

### 3.2 Gradient analysis results

**FIGURE 2:**
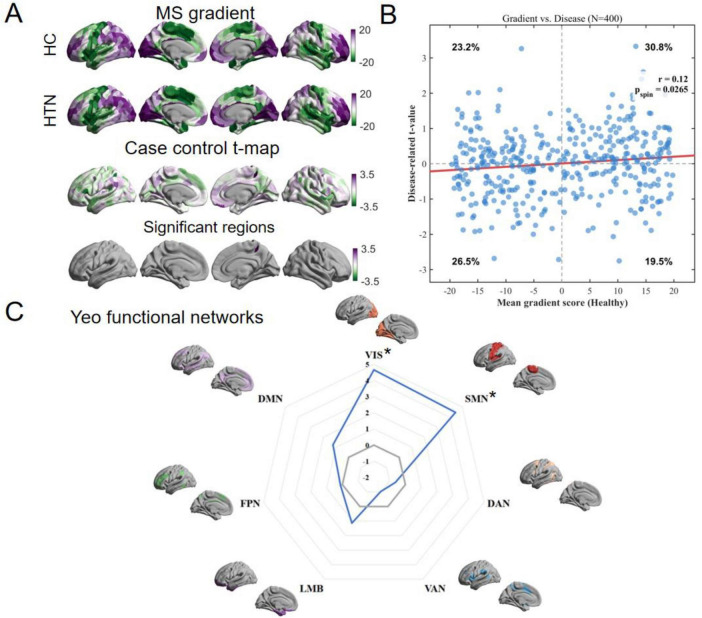
Primary morphometric similarity gradient mapping in hypertension (HTN) and healthy controls (HC) group. **(A)** The primary morphometric similarity gradient patterns in the HTN and HC groups, displaying the inter-group *t*-values and statistically significant brain regions. **(B)** The density scatterplot depicts the relationship between the baseline cortical hierarchy and the disease-related alterations. Each dot represents a brain region. The red line indicates the linear fit. **(C)** Mountain plots illustrating the distribution of the Yeo seven functional networks in patients with HTN and HC. *p < 0.05, **p < 0.01.

The principal MS gradient explained the largest variance (50%) in the MS network connectivity data, followed by the second gradient (25%). Given its dominance in capturing the global cortical hierarchy, we focused our subsequent analyses on the principal gradient as the primary axis of cortical organization. GLM analysis revealed significant inter-group differences. Following inter-individual gradient alignment (Figure 2A), regional comparisons demonstrated that gradient scores in left frontoparietal network (FPN) parietal subregion-4 (*t* = 3.190, *p* = 0.0023) and right ventral attention network (VAN) medial subregion-5 (*t* = 3.3081, *p* = 0.0016) were significantly higher in the HTN group. A significant positive spatial correlation was observed between the mean regional MS gradient map of the HC group and the case-control t-statistic map (*r* = 0.120, *p*_*spin*_ = 0.0265, Figure 2B). This spatial association reveals a global “gradient expansion” pattern: regions with negative principal MS gradients in HC tended to exhibit further decreased values in the HTN group (negative t-statistics; affecting approximately 26.5% of regions), whereas regions with positive gradients in HC tended to show increased values (positive t-statistics; affecting approximately 30.8% of regions). This “rich-get-richer” phenomenon indicates a polarization of the macroscale hierarchy in perimenopausal HTN. Furthermore, when mapped onto the Yeo 7-network functional atlas (Figure 2C), the HTN group exhibited significantly elevated gradient values within the visual network (VIS) (*t* = 4.664, *p* < 0.001) and somatomotor network (SMN) (*t* = 4.463, *p* < 0.001) networks.

### 3.3 Case-control differences in thalamic subregions

**FIGURE 3:**
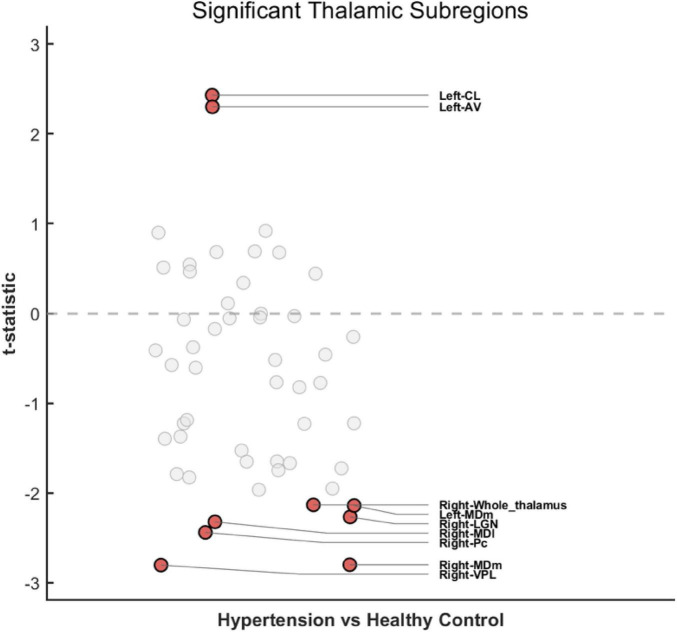
T-statistic distribution of thalamic subregion volumetric differences between hypertension (HTN) and healthy controls (HC) groups. The scatter plot illustrates the t-statistics (y-axis) derived from the general linear model (GLM) analysis for all 52 thalamic subregions. Red solid circles indicate subregions with statistically significant group differences (*p* < 0.05). Gray hollow circles represent non-significant subregions. A positive *t*-value (*t* > 0) denotes increased volume in the HTN group, whereas a negative *t*-value (*t* < 0) denotes reduced volume (atrophy) in the HTN group compared to HC.

We employed a GLM to compare the raw volumes of each thalamic subregion between patients with HTN and HCs, with TIV included as a covariate to control for head size. We observed significant volumetric reductions in seven thalamic subregions in the HTN group compared to HCs, specifically: the Right-ventral posterolateral (VPL) (*t* = −2.804, *p* = 0.007), Right-mediodorsal medial (MDm) (*t* = −2.800, *p* = 0.007), Right-paracentral (Pc) (*t* = −2.440, *p* = 0.018), Right-mediodorsal lateral (MDl) (*t* = −2.323, p = 0.024), Right-lateral geniculate nucleus (LGN) (*t* = −2.268, *p* = 0.027), Left-MDm (*t* = −2.141, *p* = 0.037), and the Right-Whole thalamus (*t* = −2.134, *p* = 0.037). Conversely, significantly increased volumes were found in two subregions: the Left-central lateral (CL) (*t* = 2.430, *p* = 0.018) and Left-anteroventral (AV) (*t* = 2.301, *p* = 0.025) (Figure 3).

### 3.4 Correlations between the altered principal MS gradient, abnormal thalamic subregions, and clinical variables

**FIGURE 4:**
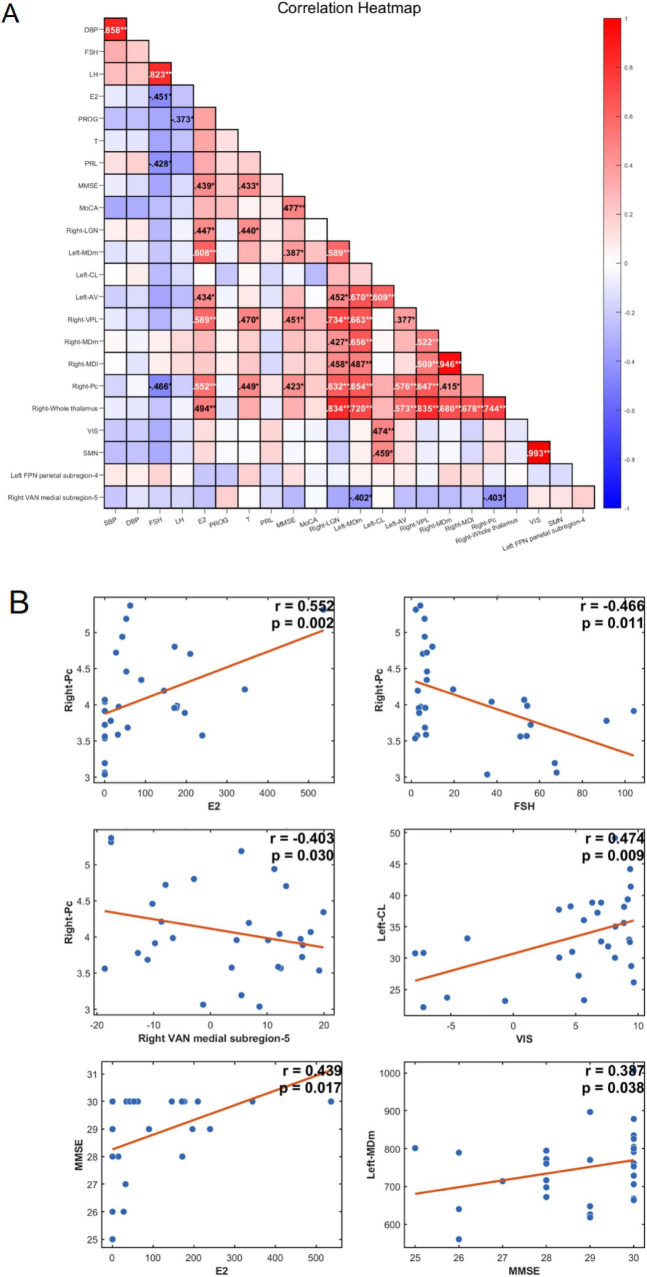
Partial correlation analysis in the hypertension (HTN) group. **(A)** The numbers represent correlation coefficients (*r*-values). A positive *r*-value denotes a positive correlation, while a negative *r*-value denotes a negative correlation (*p < 0.05, **p < 0.01). **(B)** Scatter plots depicting significant partial correlations. The plots illustrate significant associations categorized into three main groups: (1) Hormone associations: Correlations between reproductive hormones and both thalamic subregion volumes and cognitive scores. (2) Thalamus-Cognition associations: Correlations between thalamic subregion volumes and cognitive scores. (3) Thalamus-Gradient associations: Correlations between thalamic subregion volumes and functional network gradient values.

Partial correlation analysis (controlling for TIV interaction) demonstrated significant associations between reproductive hormones, thalamic subregion volumes, cognitive performance, and functional network gradients (Figure 4). Regarding hormones, E2 levels showed significant positive correlations with MMSE scores (*r* = 0.439, *p* = 0.017) and the volumes of multiple subregions, including the Left-MDm (*r* = 0.608, *p* < 0.001), Right-VPL (*r* = 0.589, *p* = 0.001), Right-Pc (*r* = 0.552, *p* = 0.002), Right-Whole_thalamus (*r* = 0.494, *p* = 0.007), Right-LGN (*r* = 0.447, *p* = 0.015), and Left-AV (*r* = 0.434, *p* = 0.019). Similarly, T levels were positively correlated with MMSE scores (*r* = 0.433, *p* = 0.019) and volumes of the Right-VPL (*r* = 0.470, *p* = 0.010), Right-Pc (*r* = 0.449, *p* = 0.015), and Right-LGN (*r* = 0.440, *p* = 0.017). In contrast, FSH levels exhibited a significant negative correlation with the Right-Pc volume (*r* = −0.466, *p* = 0.011). Regarding cognitive and network associations, MMSE scores were positively correlated with volumes of the Right-VPL (*r* = 0.451, *p* = 0.014), Right-Pc (*r* = 0.423, *p* = 0.022), and Left-MDm (*r* = 0.387, *p* = 0.038). Additionally, significant correlations were observed between thalamic subregions and functional gradients: the Left-CL volume was positively associated with both VIS (*r* = 0.474, *p* = 0.009) and SMN (*r* = 0.459, *p* = 0.012) gradients, while the Right-Pc (*r* = −0.403, *p* = 0.030) and Left-MDm (*r* = −0.402, *p* = 0.031) showed negative associations with the right VAN medial subregion-5 gradient.

### 3.5 Transcription-neuroimaging associations

Partial least squares regression analysis did not reveal significant spatial associations between gene expression profiles and the principal MS gradient alterations.

## 4 Discussion

This study demonstrates a neuroendocrine-vascular pathway where hormonal depletion and HTN interact to disrupt the brain’s hierarchy. Our findings suggest that E2 depletion and FSH elevation are linked to specific thalamic remodeling, which subsequently destabilizes cortical gradients and impairs cognition.

### 4.1 Structural foundation: bidirectional thalamic remodeling

The thalamus serves as the central gateway for sensory integration and cortical modulation (43, 44). We identified a distinct bidirectional remodeling pattern: atrophy in sensory (VPL, LGN) and executive (MDm) nuclei, juxtaposed with hypertrophy in intralaminar and anterior nuclei (CL, AV). The atrophy in VPL and LGN aligns with the “vascular vulnerability” hypothesis, suggesting that high-metabolic sensory relays are susceptible to hypertensive hypoperfusion (45, 46). The hypertrophy observed in the intralaminar and anterior nuclei (CL and AV) presents a paradoxical finding. Given the CL’s role in arousal and the AV’s role in limbic circuitry (47–49), this may represent an adaptive neuroplastic response—potentially a compensatory upregulation of thalamocortical drive to maintain cognitive homeostasis in the face of sensory deafferentation. However, we cannot rule out pathological mechanisms. In the context of hypertension, such volumetric enlargement may also be attributed to neuroinflammation, microglial activation, or interstitial edema induced by chronic hemodynamic stress. Therefore, this “hypertrophy” warrants cautious interpretation, potentially reflecting either a compensatory homeostatic effort or an active inflammatory phase of hypertensive injury, distinct from the degenerative atrophy observed in sensory nuclei.

### 4.2 Functional consequence: gradient expansion and dedifferentiation

Building upon this structural remodeling, we observed significant macroscale organizational alterations. Unlike the Default Mode Network (DMN)-specific disruption often seen in general HTN, the perimenopausal HTN group exhibited a morphological gradient expansion primarily within the VIS and SMN networks (50, 51). More specifically, localized gradient elevations were identified in the left FPN parietal subregion-4 and right VAN medial subregion-5. The alteration in the VAN parcel (right medial subregion-5) is particularly critical, as this network anchors the switching between internal and external attention (52). Furthermore, our spatial correlation analysis revealed a global “gradient expansion” pattern—where regions with negative baseline gradients became more negative, and those with positive gradients became more positive—suggesting an abnormal polarization of the entire cortical landscape. The expansion of gradients in these unimodal and attention-related regions suggests a failure of sensory-transmodal integration, often termed “functional dedifferentiation” (53, 54), indicating a breakdown of the typical sensory-transmodal hierarchy.

### 4.3 The mechanistic pathway: the “double-hit” of hormones and HTN

The hierarchical relationship between reproductive aging, structure, and function was substantiated by our correlation analyses, which elucidated the specific endocrine contributions (E2 depletion and FSH elevation) within this neuroendocrine-vascular interaction.

First, regarding the structural hierarchy: We identified a robust chain linking endocrine status to network-level disorganization. Declining E2 levels—and concurrently elevated FSH levels—were significantly correlated with atrophy of the Right-Pc. As FSH elevation is a hallmark of ovarian senescence (55), its negative correlation with Right-Pc volume strongly reinforces the link between reproductive aging and thalamic degeneration. Our results revealed a specific uncoupling: the atrophy of the Right-Pc, a key intralaminar nucleus involved in arousal and vigilance, was significantly associated with gradient expansion in the VAN. This suggests a bottom-up mechanism where the degradation of thalamic arousal inputs (from the Pc) leads to a destabilization of the cortical VAN hierarchy. This “Thalamus-VAN” disconnection may underpin the attentional deficits and difficulty in cognitive set-shifting frequently observed in hypertensive patients, providing a specific circuit-level explanation for their executive dysfunction.

Second, regarding the cognitive axis: Thalamic atrophy (specifically Left-MDm and Right-VPL) and reduced E2 levels jointly predicted lower MMSE scores. The involvement of the MDm—a critical hub for prefrontal executive loops (56, 57)—explains the multidimensional cognitive deficits.

Together, these axes support a unified model: the chronic hemodynamic stress of HTN inflicts vascular injury, while the concurrent menopausal transition (marked by E2 depletion and FSH elevation) may involve a dysregulation of neuroprotective pathways. This synergistic interaction compromises the thalamocortical integration required for both functional network stability and higher-order cognition.

Finally, our transcriptomic analysis revealed no significant spatial overlap between gradient changes and healthy gene expression profiles. The lack of significant spatial association between gradient alterations and healthy gene expression profiles suggests that the brain reorganization in perimenopausal HTN is likely driven by ‘acquired’ environmental stressors rather than being strictly constrained by the baseline genetic architecture.

### 4.4 Limitations

Several limitations of this study warrant consideration. First, the cross-sectional design precludes definitive causal inferences regarding the temporal sequence between hormonal depletion and brain remodeling; longitudinal studies are necessary to track these trajectories over time. Second, the sample size (*n* = 30 per group) was determined by the strict inclusion criteria and the available recruitment window. While this sample size is comparable to previous neuroimaging studies investigating thalamic subregions or gradients (58), it may have limited the statistical power for detecting more subtle brain-behavior associations. Future longitudinal studies with larger cohorts are needed to validate these findings. Third, we observed a distinct lateralized pattern of thalamic remodeling. Although previous research has suggested hemispheric asymmetry in vascular vulnerability or autonomic control (59, 60), the exact biological mechanisms remain to be fully elucidated in larger, more diverse cohorts. Fourth, our transcriptomic analysis relied on the AHBA database derived from healthy donors. The lack of significant spatial associations may stem from the inherent discrepancy between healthy gene expression patterns and the pathological transcriptomic alterations unique to the hypertensive perimenopausal state. Finally, the classification of perimenopause in this study was primarily based on age (45–55 years) and serum hormone markers rather than the gold-standard STRAW + 10 staging system, as longitudinal menstrual cycle diaries were not available. We acknowledge that this reliance on cross-sectional parameters may have resulted in the inclusion of individuals at slightly varying stages of reproductive aging, limiting the precision of our reproductive staging.

## 5 Conclusion

In conclusion, this study identifies a distinct neuroendocrine-vascular pathway in perimenopausal HTN, characterized by a “double-hit” of hormonal depletion and hemodynamic stress. Our findings demonstrate that the synergistic effect of estradiol loss and FSH elevation is linked to bidirectional thalamic remodeling, which in turn destabilizes the macroscale cortical hierarchy. Crucially, these structural and hierarchical disruptions—specifically the atrophy of sensory-executive thalamic nuclei and the expansion of cortical gradients—are significantly associated with diminished cognitive performance (MMSE scores). These results underscore the importance of the perimenopausal transition as a critical window for brain health. Clinical strategies should prioritize early blood pressure management alongside hormonal monitoring to safeguard the thalamocortical axis and mitigate the risk of cognitive decline in this vulnerable population.

## Data Availability

The data that support the findings of this study are not publicly available because them contain information that could compromise participant’s privacy/consent. The data are available on reasonable request from the corresponding author.

## Footnotes

1
https://surfer.nmr.mgh.harvard.edu/

2
https://fsl.fmrib.ox.ac.uk/

3
https://github.com/MICA-MNI/BrainSpace

4
https://www.github.com/netneurolab/abagen
